# Higher age and present injury at the start of the season are risk factors for in-season injury in amateur male and female football players—a prospective cohort study

**DOI:** 10.1007/s00167-023-07517-6

**Published:** 2023-08-05

**Authors:** Sofi Sonesson, Hanna Lindblom, Martin Hägglund

**Affiliations:** 1 Unit of Physiotherapy, Department of Health, Medicine and Caring Sciences, Linköping University, 581 83 Linköping, Sweden; 2 Sport Without Injury ProgrammE (SWIPE), Department of Health, Medicine and Caring Sciences, Linköping University, Linköping, Sweden

**Keywords:** Sports, Injury, Epidemiology, Knee injuries, Hip/groin injuries

## Abstract

**Purpose:**

To describe the injury prevalence, injury pattern, and potential baseline risk factors for injuries in male and female adolescent and adult amateur football players.

**Methods:**

This prospective study followed adolescent and adult amateur football players over one season March–October 2020. The study was completed by 462 players (130 men, age 20.0 ± 5.7, 14–46 years) who answered a baseline survey and a weekly web survey during the season. A total of 1456 weekly surveys were registered from males and 5041 from females. Injuries were recorded with the Oslo Sports Trauma Research Center Overuse Injury Questionnaire (OSTRC-O2). Potential baseline risk factors (age, performance of strength/conditioning training, participation in other sports, perceived importance of sporting success, self-rated training and match load, perceived balance between training/match load and recovery, previous/present injury at start of season, and injury beliefs) and their association with injury were analysed with Poisson regressions within each sex.

**Results:**

Males reported 95 injuries (262 injury weeks, weekly prevalence 18.0% (95% CI 16.1–20.1)) and females 350 injuries (1206 injury weeks, weekly prevalence 23.9% (95% CI 22.8–25.1)). Gradual-onset injuries accounted for 57% of the injuries in males and 66% in females. For males, substantial injuries were most common in the hip/groin (weekly prevalence 3.8%), ankle (2.1%), posterior thigh (2.0%), and knee (2.0%); and for females, in the knee (4.3%), ankle (2.5%), and lower leg/Achilles tendon (2.0%). Significant risk factors for injury were higher age (rate ratio males 1.05 per year increase (95% CI 1.02–1.08), females 1.03 (95% CI 1.01–1.05)), and present injury at baseline (males 1.92 (95% CI 1.27–2.89), females 1.58 (95% CI 1.19–2.09)).

**Conclusion:**

At any given week, almost one in five male and one in four female amateur football players reported new or ongoing injuries. Hip/groin injuries were more frequent in males, while female players had a higher prevalence of knee injuries. Older players and those with an existing injury at the start of the season were more prone to new injury during the season. Rehabilitation of pre-season injury and complaints are key to reduce the injury burden in amateur football.

**Level of evidence:**

Level II.

**Trial registration number** NCT04272047, Clinical trials

**Supplementary Information:**

The online version contains supplementary material available at 10.1007/s00167-023-07517-6.

## Introduction

Football is a complex, high-intensity contact sport associated with a high risk of injury [19, 21, 29–31]. Injury pattern differs with age, sex, and player level [25, 26, 31]. A higher injury incidence in older players compared to younger players has been reported in youth football [17, 27, 34, 41, 42], in adult female football [12], and in elite female football [1]. In elite male football, the association between age and injury is inconclusive with contradicting findings [3, 4, 18, 20, 22].

In elite male [4, 20], elite female [1], and in female youth football [17, 33], a history of musculoskeletal injury is associated with higher risk of future injury, especially a new injury in the same anatomical region. In elite male football, previous anterior cruciate ligament (ACL) injury has been shown to be a main risk factor for future knee injury [40].

In addition to anatomical and physiological factors, psychosocial factors have received increased attention in recent years. Personality attributes, such as trait anxiety, and a history of stressors, like high level of life stress or fear/anxiety associated with a previous injury, are positively correlated with injury rates, and psychological and social readiness for play may affect the risk of injuries in football players [32]. Perception of injury risk may be associated with risk-taking behaviour [8], which needs to be considered and taken into account in injury prevention [38].

Data on amateur football is sparse, but previous epidemiological studies on male adult players have reported lower incidence of injuries among amateur players compared to professional players [14, 36, 37]. More information is needed about the injury burden (injury prevalence, consequences, and seasonal variation) and injury pattern (injury type and injury location) in both male and female adolescent and adult amateur football. This data may form the basis for development and adaptation of injury prevention strategies in both male and female players and for various player age groups. By identifying risk factors at start of the season, preventive measures can be targeted at players who are at high risk of injury. The current aim was to describe injury prevalence, injury pattern, and potential baseline risk factors for injury in male and female adolescent and adult amateur football players.

## Materials and methods

The study was approved by the Regional Ethical Board in Linköping (Ref. no. 2017/294-31) and a later amendment by the Swedish Ethical Review Authority (Ref. no. 2019-06462).

The study design is a prospective cohort study comprising sub-analyses from a randomised trial [24]. The analyses are based on a baseline questionnaire and weekly questionnaires from amateur adolescent and adult male and female football players (14 years and older) who participated in a two-armed cluster-randomised trial with an additional non-randomised comparison arm [24]. Teams were allocated to one of two interventions: an extended *Knee Control* programme focusing on lower extremity injuries in general or an adductor strength programme focusing on groin injuries. The non-randomised comparison group was comprised of teams that already used an injury prevention exercise programme (IPEP). The study covered one 7-month season from March to October/November 2020. The study complied with the declaration of Helsinki and its later amendments.

### Study population and recruitment

Inclusion criteria were teams (1) participating in the adolescent or adult 2020 series (male 5th–8th leagues (out of 8 leagues), female 3rd–5th leagues (out of 5 leagues), male and female 16–19 series) in one regional district (Östergötland) in Sweden), (2) with at least two scheduled training sessions per week. All players 14 years and older were eligible, including players who were injured at baseline.

**Fig. 1:**
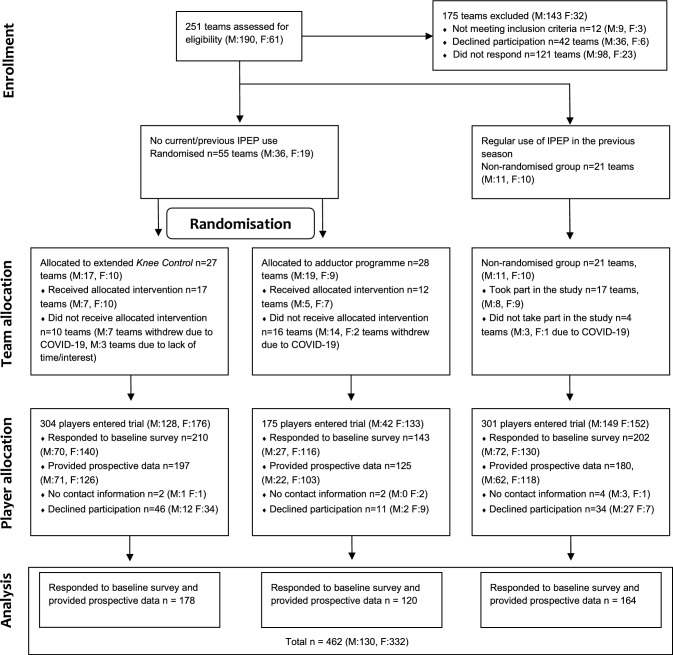
Flow diagram over the inclusion in the study. F, female; IPEP injury prevention exercise programme; and M, male

Coaches for eligible teams (*n* = 251) were approached via e-mail and telephone and received oral and written information about the study before agreeing to participate. In teams where the coach had agreed to participate, all players (and guardians for players < 15 years) then received written information about the study and were inquired for participation in the study (Fig. 1). Participation was voluntary, and individual players could decline to partake in research even though their team was involved in the study. In order to initiate the online baseline form, the player had to verify that they had read and understood the information about study participation, and subsequent response to the questionnaires was taken as consent to participate.

### Data collection

Players responded to a baseline questionnaire (Supplementary Appendix 1) at the start of the football season including questions about previous (previous season or earlier) and/or present injury in any of the locations ankle, knee, hamstrings, or groin (primary injury outcomes of the randomised trial), if they participated in other sports during the football season, if they performed strength- and/or conditioning training during the football season and questions about injury beliefs, importance of success in sports and training load (response model 7-level Likert scale): “I expect I will sustain an injury sometime during this football season” (extremely likely–extremely unlikely); “What significance does sporting success have for your interest and motivation in sports?” (little importance–great importance); “How do you rate your current total training volume?” (extremely low–extremely high); How do you rate your current total training load (quantity and intensity of training? (extremely low–extremely high); How do you rate your current match load (frequency and time on the pitch)? (extremely low–extremely high); and I have a good balance between training/match load and recovery (strongly disagree–strongly agree).

Players responded to weekly questionnaires about occurrence of injury based on the Oslo Sports Trauma Research Center Overuse Injury Questionnaire (OSTRC-O2) [10], and exposure to training and matches. Conditional branching was used to allow for targeted questions, and the players answered a minimum of 1 (no football participation due to reasons other than injury or illness, e.g. holidays) and a maximum of 28 questions (for players who had participated in football activity and had sustained a new injury) each week. Multiple injuries could be reported. When a new injury was reported, additional questions were given regarding the nature of the injury (i.e. when the injury occurred (training/match/other activity), injury location, whether it was sudden or gradual onset). In case of sudden onset, they received an additional question about the type of injury. The questionnaire covered all physical complaint injuries. All sudden-onset injuries occurring during football training or matches and all gradual-onset injuries regardless of whether symptoms first appeared during football or other activities were included. Players who reported time-loss or substantial injury to the groin or hamstrings (sudden onset or gradual onset), knee or ankle (sudden onset) were contacted via telephone by a study physiotherapist who asked about the injury and filled in a standard injury report form. Injuries were defined in line with the International Olympic Committee consensus statement [5].

All questionnaires were distributed via online software (esMakerNX3 V 3.0). The baseline questionnaires, with two reminders, were distributed after each team’s acceptance to participate, and weekly questionnaires were distributed on each Sunday evening, with reminders sent to non-responders on Tuesday and Thursday the week after.

### Data analysis

For calculation of the OSTRC severity score, the responses to each of the four questions of the OSTRC-O2 were transformed to a numerical value from 0 to 25, and these were summed to calculate a severity score from 0 to 100, where 0 represents no problems and 100 represents maximum problems [11]. Injuries were defined in line with the International Olympic Committee consensus statement [5] and covered any physical complaint injuries (irrespective of need of medical attention or time-loss), sudden- and gradual-onset injuries, time-loss injury (injury with reduced participation or absence from football training and/or matches as reported in question 1 of the OSTRC-O2), and medical attention injury (injury where the player sought medical advice or treatment). Substantial injury was defined as an injury with moderate or severe modifications in participation in football training and matches and/or moderate or severe effects on football performance, or inability to participate in football [10].

### Statistical analyses

**Fig. 2:**
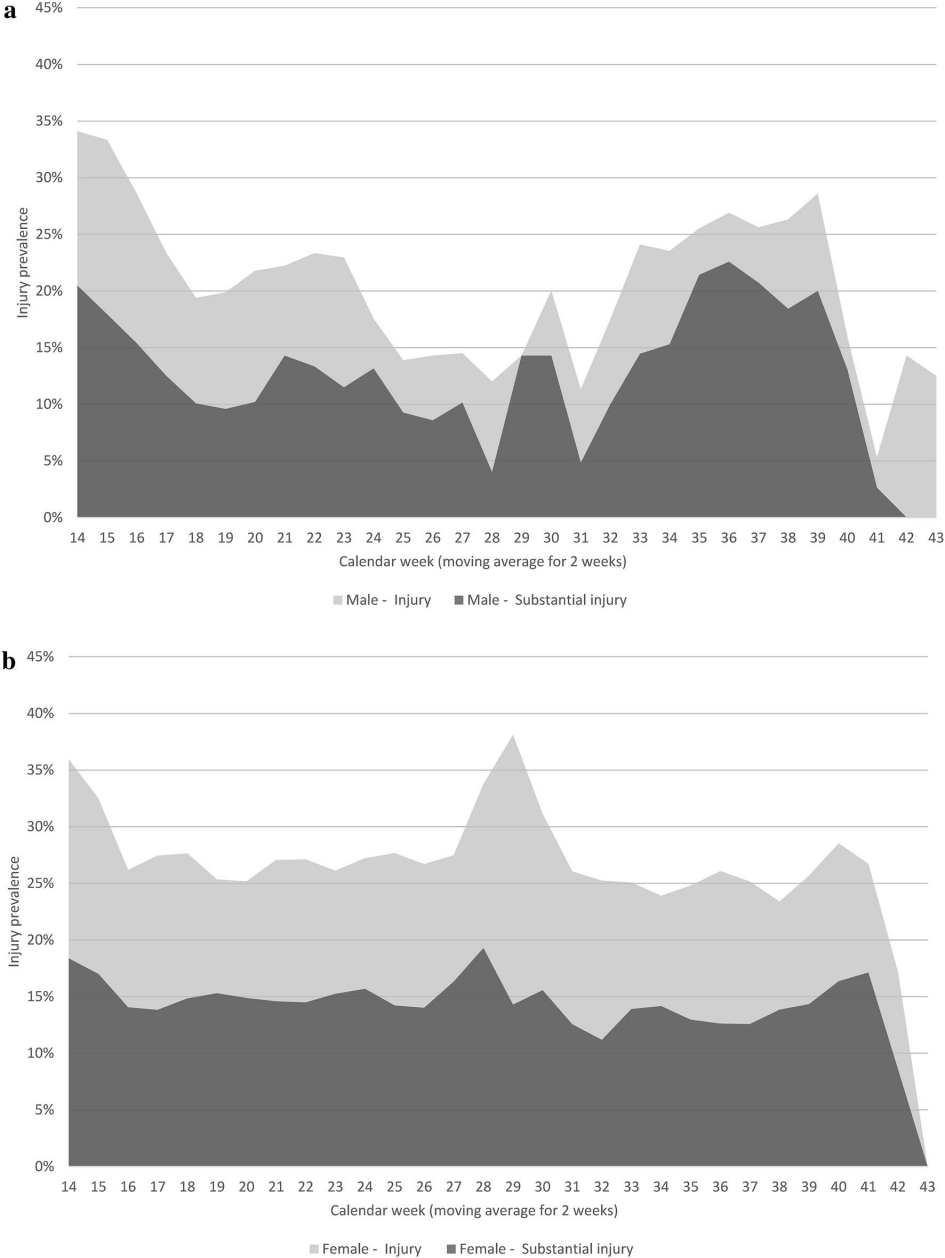
Weekly injury prevalence (moving average for 2 weeks*) for **a** male and **b** female amateur football players. *Average of the current week and the preceding week. Many teams had summer break during weeks 27–29

All analyses were conducted for male and female players separately. Descriptive statistics are presented with sum, mean ± standard deviation, and *n* (%). Weekly injury prevalence was calculated as the number of player reports where a player reported new or ongoing injury divided by the total number of eligible player reports that same week. Weekly injury prevalences are displayed as a moving average for 2 consecutive weeks, i.e. data for a certain week is the average of the current week and the preceding week (Fig. 2). Seasonal injury prevalence and substantial injury prevalence are presented with 95% confidence intervals (CI) (Table 1).

**Table 1:** Descriptive statistics of weekly reports during season

	Male (*n* = 130)		Female (*n* = 332)	
	Sum*	Mean ± SD	Sum*	Mean ± SD
Registered weeks, per player season	1456	11.2 ± 7.5	5041	15.2 ± 7.9
Training hours, per player week	4399	3.1 ± 1.6	13,314	2.6 ± 1.2
Match hours, per player week	778	0.5 ± 0.4	3106	0.5 ± 0.4
Exposure hours, per player week	5177	3.6 ± 1.8	16,421	3.2 ± 1.5
Injuries, per player season^†^	95	1.3	350	1.4
Injury incidence, per 1000 h exposure^§^		18.4 (15.0–22.4)		21.3 (19.2–23.7)
Time-loss injuries, per player season^†^	57	0.8	198	0.8
Time-loss injury incidence, per 1000 h exposure^§^		11.0 (8.5–14.3)		12.1 (10.5–13.9)
Total injury weeks, per player season^†^	262	3.6	1206	4.8
Injury prevalence^§^ (%)		18.0 (15.9–20.3)		23.9 (22.6–25.3)
Substantial injury weeks, per player season^†^	162	2.2	653	2.6
Substantial injury prevalence^§^ (%)		11.1 (9.5–13.0)		13.0 (12.0–14.0)

SD, standard deviation

*Sum of all players during season; ^†^Based on a 20-week season; and ^§^95% confidence interval

Injury incidence rates were calculated as total number of injuries divided by total exposure hours. Both new injuries and re-injuries were included, but injuries that were persistent from baseline were excluded. Seasonal injury incidence rates and time-loss incidence rates are presented as injuries per 1000 h exposure with 95% CI (Table 1).

Potential baseline risk factors (age, strength- and/or conditioning training beside regular football training, participation in other sports during football season, perceived importance of sporting success, self-rated training and match load, balance between training/match load and recovery, previous/present injury at start of the season, injury beliefs) for injury were analysed with Poisson regressions. The basic model included the number of new injuries during the season as the target variable, the natural logarithm of total exposure hours during the season as the offset variable, team as a random factor using variance component as covariance structure, and intervention group (extended *Knee Control*, Adductor group, Comparison group) as a fixed factor. Log link function was used in the models to estimate the risk of injury. The analyses were performed in two steps, based on the basic model. In step 1, each risk factor was entered as a fixed factor in the basic model. In step 2, significant risk factors from step 1 were entered in a final multivariable model (Supplementary Appendix 2a and 2b). Variance inflation factor (VIF) of each potential risk factor was calculated to check for multi-collinearity in the multivariable model (VIF values, range 1.1–2.1). As a sensitivity analysis, we analysed potential baseline risk factors for the total cohort and tested for two-way interactions of player sex and each risk factor. The level of significance was set at *p* < 0.05.

IBM SPSS Statistics for Windows (V28.0. Armont, New York) was used for all analyses. An experienced statistician was responsible for preparing the database and data analyses.

An a priori sample size calculation was made for the randomised trial [24], while no sample size calculation was made for the current analyses.

## Results

In total, 462 players (130 males and 332 females) responded to the baseline questionnaire and to weekly questionnaires during the season and were included in the analyses. There were 1456 registered weeks for male players and 5041 registered weeks for female players (Table 1).

Mean age was 20.6 ± 6.0 years for males and 19.9 ± 5.7 years for females. Players’ injury beliefs, perceived importance of sporting success, and estimation of workload are displayed in Supplementary Appendix 1. Average weekly exposure during the season (football training and matches) was 3.6 ± 1.8 h for males and 3.2 ± 1.5 h for females (Table 1).

### Injury burden

Previous or present injury at baseline was reported by 77.7% of male players and 79.2% of female players. Males reported a total of 95 injuries spread over 262 injury weeks, weekly prevalence 18.0% (95% CI 15.9–20.3) (Table 1). Based on a 20-week season and a squad of 15 players, there would be 20 injuries (12 time-loss) and 54 total injury weeks (33 substantial injury weeks) per team-season. Females reported a total of 350 injuries spread over 1206 injury weeks, weekly prevalence 23.9% (95% CI 22.6–25.3) (Table 1). Based on a 20-week season and a squad of 15 players, there would be 21 injuries (12 time-loss) and 72 total injury weeks (39 substantial injury weeks) per team-season. Weekly injury prevalence during the season with moving average for 2 consecutive weeks is displayed in Fig. 2.

### Injury pattern

Gradual-onset injuries accounted for 57.3% of the injuries in males and 65.9% in females. Among sudden-onset injuries, joint sprain/dislocation represented 17.6% of all injuries in males and 21.0% in females, while muscle injury accounted for 21.8% and 9.4%, respectively (Table 2).

**Table 2:** Injury locations and injury types in male and female amateur football players

Injury locations	Sex	Injury types						Prevalence weeks
		Sudden onset					Gradual onset	
		Contusion	Fracture	Joint sprain/ dislocation	Muscle injury	Other sudden onset		
		*n* (%)	*n* (%)	*n* (%)	*n* (%)	*n* (%)	*n* (%)	*n*
Head/neck	Male	0 (0%)	0 (0%)	0 (0%)	0 (0%)	0 (0%)	1 (100%)	1
	Female	0 (0%)	0 (0%)	0 (0%)	0 (0%)	26 (54.2%)	22 (45.8%)	48
Trunk	Male	1 (14.3%)	0 (0%)	0 (0%)	4 (57.1%)	0 (0%)	2 (28.6%)	7
	Female	8 (9.6%)	0 (0%)	0 (0%)	5 (6%)	2 (2.4%)	68 (81.9%)	83
Upper extremity	Male	0 (0%)	0 (0%)	6 (85.7%)	0 (0%)	1 (14.3%)	0 (0%)	7
	Female	4 (11.8%)	5 (14.7%)	10 (29.4%)	4 (11.8%)	2 (5.9%)	9 (26.5%)	34
Hip/groin	Male	0 (0%)	0 (0%)	0 (0%)	6 (7.5%)	0 (0%)	74 (92.5%)	80
	Female	0 (0%)	0 (0%)	0 (0%)	24 (24.2%)	0 (0%)	75 (75.8%)	99
Posterior thigh	Male	1 (2.4%)	0 (0%)	*N/A*	32 (78.0%)	0 (0%)	8 (19.5%)	41
	Female	1 (1.0%)	0 (0%)	*N/A*	43 (42.6%)	0 (0%)	57 (56.4%)	101
Anterior thigh	Male	1 (4.8%)	0 (0%)	*N/A*	12 (57.1%)	0 (0%)	8 (38.1%)	21
	Female	8 (12.1%)	0 (0%)	*N/A*	32 (48.5%)	0 (0%)	26 (39.4%)	66
Knee	Male	2 (5.0%)	0 (0%)	8 (20.0%)	*N/A*	6 (15.0%)	24 (60.0%)	40
	Female	20 (5.8%)	0 (0%)	115 (33.1%)	*N/A*	2 (0.6%)	210 (60.5%)	347
Lower leg/Achilles tendon	Male	1 (6.7%)	0 (0%)	*N/A*	3 (20.0%)	0 (0%)	11 (73.3%)	15
	Female	1 (0.6%)	2 (1.2%)	*N/A*	5 (3.1%)	0 (0%)	154 (95.1%)	162
Ankle	Male	3 (6.1%)	0 (0%)	32 (65.3%)	0 (0%)	0 (0%)	14 (28.6%)	49
	Female	28 (12.3%)	0 (0%)	126 (55.3%)	0 (0%)	1 (0.4%)	73 (32.0%)	228
Foot/toe	Male	3 (23.1%)	1 (7.7%)	0 (0%)	0 (0%)	2 (15.4%)	7 (53.8%)	13
	Female	15 (13.0%)	1 (0.9%)	2 (1.7%)	0 (0%)	4 (3.5%)	93 (80.9%)	115
Unspecified location	Male	0 (0%)	0 (0%)	0 (0%)	0 (0%)	0 (0%)	1 (100%)	1
	Female	1 (11.1%)	0 (0%)	0 (0%)	0 (0%)	0 (0%)	8 (88.9%)	9
All locations	Male	12 (4.6%)	1 (0.4%)	46 (17.6%)	57 (21.8%)	9 (3.4%)	150 (57.3%)	262
	Female	86 (7.1%)	8 (0.7%)	253 (21.0%)	113 (9.4%)	37 (3.1%)	795 (65.9%)	1206

N/A, not applicable

**Fig. 3:**
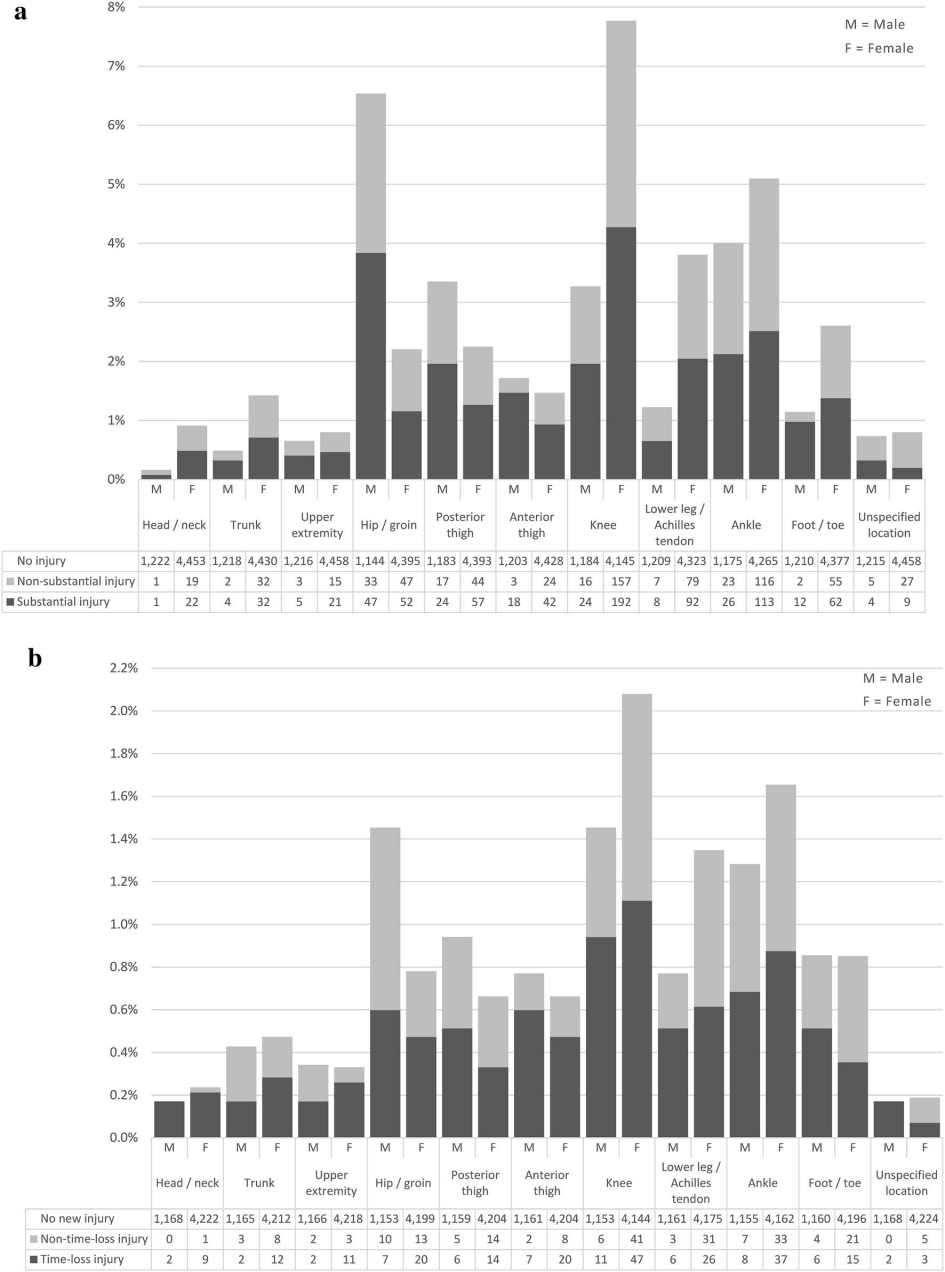
**a** Injury prevalence weeks with substantial and non-substantial injuries in each location for male and female amateur football players. **b** Injury incidence weeks with new time-loss and non-time-loss injuries in each location for male and female amateur football players

**Fig. 4:**
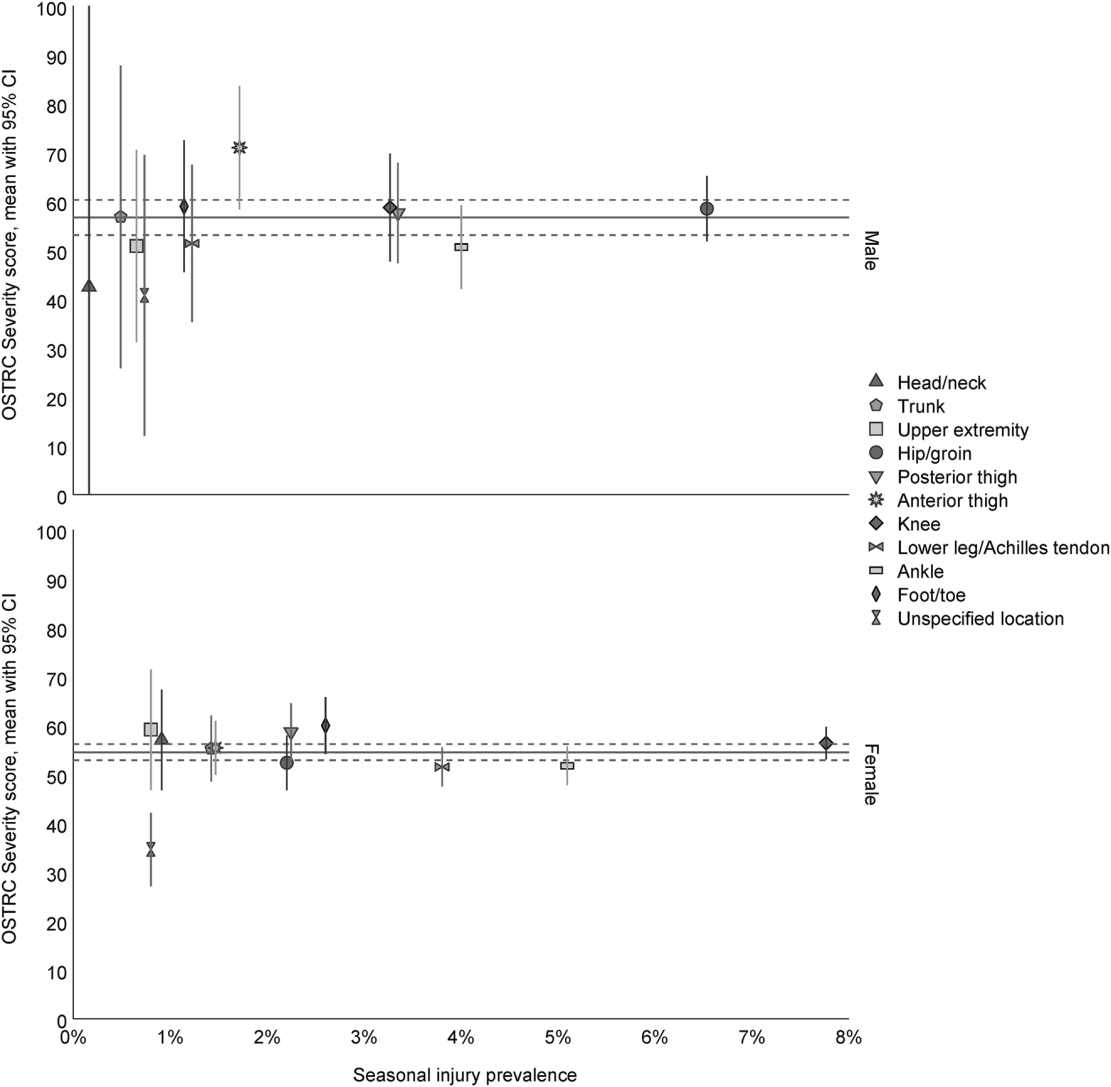
Seasonal injury prevalence by location displayed in relation to the OSTRC severity score, for male and female amateur football players. The horizontal line represents the mean OSTRC severity score with 95% CI (dotted lines) for all injuries

The hip/groin was the most injured body location in male players, and the knee in females. Substantial injuries were most common in males in the hip/groin, ankle, posterior thigh, and knee, and in females in the knee, ankle, and lower leg/Achilles tendon (Fig. 3a). Time-loss injuries were most common in males in the knee, ankle, hip/groin, and anterior thigh, and in females in the knee, ankle, and lower leg/Achilles tendon (Fig. 3b). A matrix of the seasonal injury prevalence for each body location in relation to the OSTRC severity score is displayed in Fig. 4.

### Risk factors for injury

Step 1, basic model: For males, age was a significant risk factor for injury, with 7% increased incidence for each year of age increase. Present injury at the start of the season increased the risk of injury twofold. Estimation of present training volume was associated with injury incidence with 15% reduced injury rate for each unit of change towards high training volume (Table 3).

**Table 3:** Potential baseline risk factors for injury in male and female amateur football players

Potential baseline risk factors	Males					Females				
	*n*	*p* value	exp(beta)	95% CI for exp(beta)		*n*	*p* value	exp(beta)	95% CI for exp(beta)	
				Lower	Upper				Lower	Upper
Age	130	< 0.001	1.066	1.035	1.097	332	< 0.001	1.034	1.018	1.051
Age, interval										
14–16 years*	37	< 0.001				92	0.001			
17–19 years	40	n.s	1.025	0.684	1.535	134	n.s	1.105	0.795	1.537
20–24 years	29	n.s	1.429	0.690	2.963	53	n.s	0.999	0.711	1.403
25–29 years	10	0.000	2.778	1.672	4.617	22	0.031	1.623	1.046	2.519
30–46 years	14	0.001	3.309	1.645	6.655	31	0.002	1.934	1.266	2.956
Strength/conditioning training beside regular football training										
No	12					40				
Yes	118	n.s	1.064	0.733	1.545	292	n.s	0.817	0.656	1.017
Other sports during football season										
No	116					268				
Yes	14	n.s	1.498	0.760	2.954	64	n.s	1.174	0.805	1.710
Previous and/or present injury^†^										
No previous or present injury*	29	< 0.001					0.001			
Previous injury, no present injury	48	n.s	0.982	0.494	1.950	122	0.026	1.492	1.050	2.119
Present injury, with or without previous injury	53	0.005	2.104	1.258	3.520	141	< 0.001	1.751	1.316	2.331
Injury beliefs during season^a^	126	n.s	0.848	0.693	1.038	329	0.006	0.931	0.885	0.980
Importance of sporting success on motivation to do sports^b^	127	n.s	0.974	0.799	1.189	328	n.s	0.985	0.898	1.081
Estimation of present training volume^c^	130	0.021	0.849	0.740	0.975	332	n.s	0.951	0.861	1.051
Estimation of present training load^c^	130	n.s	0.917	0.785	1.072	332	n.s	0.997	0.907	1.097
Estimation of present match load^c^	130	n.s	0.884	0.693	1.127	332	n.s	0.954	0.881	1.033
Good balance between training/match load and recovery^d^	130	n.s	1.094	0.905	1.321	332	n.s	0.958	0.876	1.048

Step 1, Based on Poisson regression on number of new injuries during the season, with the natural logarithm of total exposure hours during the season entered as an offset, and with team entered as a random factor, using variance component as covariance structure, and intervention entered as a fixed factor in addition to each potential baseline risk factor; CI, confidence interval; exp(beta), risk estimate

*p value of main fixed effect; ^†^Ankle, knee, posterior thigh, or hip/groin injury

^a^1 = extremely likely, 7 = extremely unlikely; ^b^1 = little importance, 7 = great importance; ^c^1 = extremely low, 7 = extremely high; ^d^1 = strongly disagree, 7 = strongly agree

For females, age was a significant risk factor for injury, with 3% increased incidence for each year of age increase. Previous injury and present injury at start of the season increased the risk of injury 1.5 times and 1.8 times, respectively. Injury beliefs were associated with injury incidence with 7% reduced injury rate for each unit of change towards a belief that it is unlikely to sustain an injury (Table 3).

Step 2, multivariable model: For both males and females, only age and present injury were still significant in the multivariable model (Supplementary Appendix 2).

The sensitivity analysis of baseline risk factors for the total cohort showed that age, present injury at baseline, and estimation of present training volume were significant risk factors, with no evident difference between male and female players according to the non-significant two-way interactions of players’ sex and each risk factor (Supplementary Appendix 3).

## Discussion

The main findings were that almost one in five male amateur football players and one in four female amateur football players reported presence of an injury at a given week. The hip/groin was the most injured anatomical region in males, and the knee in females. Older players (≥ 25 years) and players who had had an ongoing injury at the start of season had an increased risk of a new injury during the season.

The high proportion of injuries in the lower extremity is in line with previous research in football [16, 25, 26, 31, 36]. The hip/groin was the most injured region in the current male cohort, which contrasts with previous research on male youth players where thigh injury was most common, followed by ankle, and knee, and the hip/groin was only the fourth most common area [31]. Also in professional male football, the hip/groin is the fourth most common injury location, and the injury incidence is highest in the thigh, followed by the knee and ankle [26]. Groin injuries were more frequent in men compared to women, which is in line with previous research [23, 39]. In the current female cohort, the knee and ankle were the most injured locations, which is consistent with previous results on youth [31], adult female [2, 12, 25], and elite female [23] players, while another study in elite female football found that the most commonly injured locations were the ankle and hamstrings [15].

The total (23.9 vs 18.0%) and substantial (13.0 vs 11.1%) weekly injury prevalence was slightly higher for female players. A higher rate of more severe injuries in female football players compared to their male counterparts [25], as well as a higher risk of ACL injury in female players [9], has been reported in previous studies. Differences in injury characteristics between male and female players may be attributed to different anthropometrics and physical characteristics between sexes [30], but also to differences in training history and cultural aspects in sports [28].

Higher age and previous and present injury at the start of the season were identified as risk factors for injury during the season, which tallies with previous studies. Higher injury incidence in older players has been reported in youth football [17, 34, 41, 42], in adult female football [12], in elite female football [1], while studies in male elite football show conflicting results [4, 18, 20, 22]. A history of musculoskeletal injury is associated with higher risk of future injury in elite male [4, 20] and female [1] players, especially a new injury in the same anatomical location in the previously injured limb [1]. In youth female football, players with knee complaints pre-season are at higher risk of acute knee injury during the season [17]. Previous ACL injury and other knee injuries are major risk factors for ACL injury [13, 35]. Clinical implications based on previous research and the present study results are that players with previous injury and players with injury at the start of the season particularly could benefit from extended rehabilitation, injury prevention exercises, and/or individual adjustment in training load.

Injury beliefs were associated with injury incidence with 7% reduced injury rate for each unit of change towards a belief that it is unlikely to sustain an injury. This could be interpreted as the player’s perception of injury risk was adequate, i.e. the players could make a reasonable estimation of their risk of injury. This contrasts with previous research suggesting that perception of low injury risk could be associated with a risk-taking behaviour [8] and thus make the player more prone to injury. Only 29% of the players (male and female) in the present cohort believed it was likely that they would sustain an injury during the season (1–3 on the Likert scale). This is considerably lower compared to a recent systematic review that reported that most football players believed that their risk of sustaining an injury was moderate to high, and players expected to sustain at least one injury over the course of the following season [8]. Also, compared to players’ expectations at the beginning of the season, where 29% of players believed it was likely that they would sustain an injury during the season, the injury burden was substantially higher, as 130 male players reported a total of 95 injuries, and the 332 female players reported a total of 350 injuries during the season.

### Study strengths and limitations

A strength of the study is that a validated questionnaire previously used in sport surveillance was used to capture injuries [10, 11]. However, the baseline questionnaire containing questions about potential risk factors consisted of single Assessment Numeric Evaluation (SANE) questions that has not been validated and detailed information about the constructs cannot be determined. Though, in general, single-item scales have acceptable psychometric properties [43] and were appropriate in this prospective study with repeated surveys to limit response burden. Data are self-reported which entails uncertainty as to the validity of injury and exposure data. However, training and match exposure and injuries were reported weekly to limit recall bias. Moreover, players who reported time-loss or substantial injury to the groin or hamstrings (sudden onset or gradual onset), knee or ankle (sudden onset) were contacted via telephone by a study physiotherapist who asked about the injury and filled in a standard injury report form to validate the injury.

Other factors than those assessed in the current study may contribute to the injury risk. Risk factors for injury are complex and multifactorial, and a player’s injury profile depends on various internal risk factors and exposure to external risk factors [6, 7].

The current study is a secondary analysis of a randomised trial where teams were allocated to one of two interventions: an extended *Knee Control* programme focusing on lower extremity injuries in general or an adductor programme focusing on groin injuries. The non-randomised comparison group comprised teams that already used an injury prevention exercise programme (IPEP). All players were included in the analyses, and potential risk factors were calculated with Poisson regression with team entered as a random factor and intervention group entered as a fixed factor to account for the potential influence of team and group. All analyses were stratified within each sex to allow for interpretation of implication of study results in male and female teams, respectively. The male cohort is rather small (130 males versus 332 females); consequently, the results need to be interpreted with caution. The study was carried out during the COVID-19 pandemic, and the competitive season was postponed from April to June. It is unclear whether our findings can be generalized to a normal season. Since all teams in the present study used an IPEP, the injury burden is probably lower than among comparable teams on the same playing level that do not regularly perform an IPEP.

Clinical implications of the present study are that based on the considerable injury burden in amateur football, preventive measures should have high priority and injury-risk mitigation strategies may be differentiated in male and female teams, and also based on individual player characteristics. Due to differences in injury patterns between sexes, the primary target areas for IPEPs could be somewhat different between male and female amateur football players, i.e. more emphasis on the hip/groin in males, and emphasis on the knee in females. Moreover, since previous injury is a significant risk factor for injury during the season, a plausible interpretation is that rehabilitation after injuries might be insufficient, and players may start the season not fully physically and/or mentally prepared for play. Amateur football players with previous injury or present injury at the start of the season might need special attention from the coach and/or medical personnel to achieve individually adapted exercises and adjustment in training and/or workload. These adaptations may be of particular importance for older players.

## Conclusion

At any given week, almost one in five male amateur football players and one in four female amateur football players reported new or ongoing injuries. Hip/groin injuries were more frequent in males, while female players had a higher prevalence of knee injuries. Older players and those with an existing injury at the start of the season were more prone to new injury during the season. Rehabilitation of pre-season injury and complaints are key to reduce the injury burden in amateur football.

## Supplementary Information

Below is the link to the electronic supplementary material.

Supplementary file1 (DOCX 35 KB)

## Data Availability

Deidentified data are available from the authors upon reasonable request.
