# Supplementary material for: Higher age and present injury at the start of the season are risk factors for in-season injury in amateur male and female football players—a prospective cohort study
Source: Knee Surg Sports Traumatol Arthrosc. 2023 Aug 5;31(10):4618–30. doi: 10.1007/s00167-023-07517-6 (PMC10471640; doi:10.1007/s00167-023-07517-6)
Supplement: Supplementary file 1 — Supplementary file1 (DOCX 35 KB) [file 167_2023_7517_MOESM1_ESM.docx]

Appendix 1. Descriptive statistics of questions from baseline questionnaire to players.

|  | Male (n = 130) | | Female (n = 332) | |
| --- | --- | --- | --- | --- |
|  | Mean ± SD | n (%) | Mean ± SD | n (%) |
| Extended *Knee Control* |  | 56 (43.1%) |  | 122 (36.7%) |
| Adductor group |  | 21 (16.2%) |  | 99 (29.8%) |
| Comparison group |  | 53 (40.8%) |  | 111 (33.4%) |
| Age | 20.6 ± 6.0 |  | 19.9 ± 5.7 |  |
| *14–16 years* |  | 37 (28.5%) |  | 92 (27.7%) |
| *17–19 years* |  | 40 (30.8%) |  | 134 (40.4%) |
| *20–24 years* |  | 29 (22.3%) |  | 53 (16.0%) |
| *25–29 years* |  | 10 (7.7%) |  | 22 (6.6%) |
| *30–46 years* |  | 14 (10.8%) |  | 31 (9.3%) |
| Strength/or conditioning training beside regular football training |  | 118 (90.8%) |  | 292 (88.0%) |
| Other sports during football season |  | 14 (10.8%) |  | 64 (19.3%) |
| *Do you have a previous and/or present injury?^*^* |  |  |  |  |
| *no previous or present injury* |  | 29 (22.3%) |  | 69 (20.8%) |
| *previous injury* |  | 95 (73.1%) |  | 233 (70.2%) |
| *present injury* |  | 53 (40.8%) |  | 141 (42.5%) |
| *present injury, without previous injury* |  | 6 (4.6%) |  | 30 (9.0%) |
| *present injury, with previous injury* |  | 47 (36.2%) |  | 111 (33.4%) |
| *I expect I will sustain an injury sometime during this football season* | 4.4 ± 1.5 |  | 4.3 ± 1.5 |  |
| *extremely likely 1* |  | 4 (3.2%) |  | 14 (4.3%) |
| *very likely 2* |  | 8 (6.3%) |  | 26 (7.9%) |
| *quite likely 3* |  | 25 (19.8%) |  | 53 (16.1%) |
| *neither likely nor unlikely 4* |  | 32 (25.4%) |  | 85 (25.8%) |
| *quite unlikely 5* |  | 23 (18.3%) |  | 72 (21.9%) |
| *very unlikely 6* |  | 27 (21.4%) |  | 52 (15.8%) |
| *extremely unlikely 7* |  | 7 (5.6%) |  | 27 (8.2%) |
| *What significance does sporting success have for your interest and motivation in sports?* | 4.9 ± 1.6 |  | 4.8 ± 1.6 |  |
| *little importance 1* |  | 6 (4.7%) |  | 18 (5.5%) |
| *2* |  | 6 (4.7%) |  | 18 (5.5%) |
| *3* |  | 10 (7.9%) |  | 29 (8.8%) |
| *4* |  | 21 (16.5%) |  | 68 (20.7%) |
| *5* |  | 37 (29.1%) |  | 71 (21.6%) |
| *6* |  | 25 (19.7%) |  | 75 (22.9%) |
| *great importance 7* |  | 22 (17.3%) |  | 49 (14.9%) |
| *How do you rate your current total training volume?* | 4.6 ± 1.1 |  | 4.6 ± 1.1 |  |
| *extremely low 1* |  | 0 (0%) |  | 2 (0.6%) |
| *very low 2* |  | 4 (3.1%) |  | 12 (3.6%) |
| *quite low 3* |  | 13 (10.0%) |  | 31 (9.3%) |
| *neither low nor high 4* |  | 42 (32.3%) |  | 92 (27.7%) |
| *quite high 5* |  | 47 (36.2%) |  | 124 (37.3%) |
| *very high 6* |  | 16 (12.3%) |  | 64 (19.3%) |
| *extremely high 7* |  | 8 (6.2%) |  | 7 (2.1%) |
| *How do you rate your current total training load (quantity and intensity of training?* | 4.5 ± 1.1 |  | 4.7 ± 1.1 |  |
| *extremely low 1* |  | 1 (0.8%) |  | 4 (1.2%) |
| *very low 2* |  | 3 (2.3%) |  | 9 (2.7%) |
| *quite low 3* |  | 18 (13.8%) |  | 34 (10.2%) |
| *neither low nor high 4* |  | 39 (30.0%) |  | 80 (24.1%) |
| *quite high 5* |  | 43 (33.1%) |  | 135 (40.7%) |
| *very high 6* |  | 23 (17.7%) |  | 64 (19.3%) |
| *extremely high 7* |  | 3 (2.3%) |  | 6 (1.8%) |
| *How do you rate your current match load (frequency and time on the pitch)?* | 3.8 ± 1.5 |  | 4 ± 1.6 |  |
| *extremely low 1* |  | 12 (9.2%) |  | 37 (11.2%) |
| *very low 2* |  | 17 (13.1%) |  | 27 (8.2%) |
| *quite low 3* |  | 14 (10.8%) |  | 48 (14.5%) |
| *neither low nor high 4* |  | 45 (34.6%) |  | 85 (25.7%) |
| *quite high 5* |  | 30 (23.1%) |  | 67 (20.2%) |
| *very high 6* |  | 10 (7.7%) |  | 59 (17.8%) |
| *extremely high 7* |  | 2 (1.5%) |  | 8 (2.4%) |
| *I have a good balance between training/match load and recovery* | 4.9 ± 1.2 |  | 5.1 ± 1.4 |  |
| *strongly disagree 1* |  | 1 (0.8%) |  | 4 (1.2%) |
| *2* |  | 2 (1.5%) |  | 14 (4.2%) |
| *3* |  | 15 (11.5%) |  | 35 (10.5%) |
| *neither disagree nor agree 4* |  | 23 (17.7%) |  | 34 (10.2%) |
| *5* |  | 45 (34.6%) |  | 86 (25.9%) |
| *6* |  | 32 (24.6%) |  | 121 (36.4%) |
| *strongly agree 7* |  | 12 (9.2%) |  | 38 (11.4%) |
| *SD, standard deviation*  ^*^ *Ankle, knee, hamstrings, or groin injury* | | | | |

Appendix 2. Potential baseline risk factors for injury in male and female amateur football players, final multivariable model.

|  | | |  | Males | | | |  | Females | | | |
| --- | --- | --- | --- | --- | --- | --- | --- | --- | --- | --- | --- | --- |
| Potential baseline risk factors | | | n | p-value | exp(beta) | 95% CI for exp(beta) | |  | p-value | exp(beta) | 95% CI for exp(beta) | |
|  |  |  |  |  |  | lower | upper | n |  |  | lower | upper |
| Age | | | 130 | 0.001 | 1.048 | 1.019 | 1.078 | 332 | <0.001 | 1.029 | 1.012 | 1.046 |
| Previous and/or present injury ^†^ | | *no previous or present injury*^*^ | 29 | <0.001 |  |  |  | 69 | 0.006 |  |  |  |
| *previous injury, no present injury* | | | 48 | n.s | 1.051 | 0.587 | 1.882 | 122 | 0.061 | 1.393 | 0.984 | 1.972 |
| *present injury, with or without previous injury* | | | 53 | 0.002 | 1.916 | 1.272 | 2.888 | 141 | 0.001 | 1.581 | 1.194 | 2.093 |
| Injury beliefs during season ^a^ | | | 126 |  |  |  |  | 329 | 0.065 | 0.954 | 0.908 | 1.003 |
| Estimation of present training volume ^c^ | | | 130 | n.s | 0.858 | 0.735 | 1.003 | 332 |  |  |  |  |
|  | *Step 2, Final multivariable model, based on significant variables from Step 1 which is displayed in table 3; The following variables were not included in the multivariable model Age (interval), Strength/conditioning training beside regular football training, Other sports during football season, Importance of sporting success on motivation to do sports, Estimation of present training load, Estimation of present match load, Good balance between training/match load and recovery; CI, confidence interval; exp(beta), risk estimate.*  ^*^ *p-value of main fixed effect;* ^†^ *Ankle, knee, posterior thigh, or hip/groin injury*  ^a^ *1= extremely likely, 7= extremely unlikely*; ^b^ *1= little importance, 7= great importance*; ^c^ *1= extremely low, 7= extremely high*; ^d^ *1= strongly disagree, 7= strongly agree* | | | | | | | | | | | |

Appendix 3. Potential baseline risk factors for injury, in male and female amateur football players.^*^

| Potential baseline risk factors | n | p-value | exp(beta) | 95% CI for exp(beta) | |
| --- | --- | --- | --- | --- | --- |
|  |  |  |  | lower | upper |
| Age | 462 | <0.001 | 1.065 | 1.036 | 1.095 |
| Sex, female | 462 | n.s | 2.007 | 0.963 | 4.183 |
| Interaction, Age × Sex, female |  | n.s | 0.974 | 0.944 | 1.005 |
| Strength/cardio training beside regular football training |  |  |  |  |  |
| *no* | 52 |  |  |  |  |
| *yes* | 410 | n.s | 1.024 | 0.739 | 1.418 |
| Sex, female |  | n.s | 1.296 | 0.857 | 1.960 |
| Interaction, Factor × Sex, female |  | n.s | 0.796 | 0.539 | 1.177 |
| Other sports during football season |  |  |  |  |  |
| *no* | 384 |  |  |  |  |
| *yes* | 78 | n.s | 1.455 | 0.785 | 2.698 |
| Sex, female |  | n.s | 1.074 | 0.748 | 1.542 |
| Interaction, Factor × Sex, female |  | n.s | 0.806 | 0.391 | 1.661 |
| Previous and/or present injury ^†^ |  |  |  |  |  |
| *no previous or present injury* ^§^ | 98 | <0.001 |  |  |  |
| *previous injury, no present injury* | 170 | n.s | 1.266 | 0.516 | 3.105 |
| *present injury, with or without previous injury* | 194 | 0.008 | 2.562 | 1.277 | 5.139 |
| Sex, female |  | n.s | 1.238 | 0.538 | 2.852 |
| Interaction, Factor × Sex, female |  | n.s |  |  |  |
| *previous injury, no present injury* |  | n.s | 1.180 | 0.450 | 3.089 |
| *present injury, with or without previous injury* |  | n.s | 0.684 | 0.323 | 1.450 |
| Injury beliefs during season ^a^ | 455 | n.s | 0.848 | 0.693 | 1.037 |
| Sex, female |  | n.s | 0.813 | 0.339 | 1.947 |
| Interaction, Factor × Sex, female |  | n.s | 1.099 | 0.893 | 1.351 |
| Importance of sporting success on motivation to do sports ^b^ | 455 | n.s | 0.956 | 0.787 | 1.163 |
| Sex, female |  | n.s | 0.922 | 0.292 | 2.914 |
| Interaction, Factor × Sex, female |  | n.s | 1.031 | 0.830 | 1.280 |
| Estimation of present training volume ^c^ | 462 | 0.004 | 0.841 | 0.749 | 0.945 |
| Sex, female |  | n.s | 0.598 | 0.260 | 1.371 |
| Interaction, Factor × Sex, female |  | n.s | 1.130 | 0.969 | 1.317 |
| Estimation of present training load ^c^ | 462 | n.s | 0.905 | 0.791 | 1.037 |
| Sex, female |  | n.s | 0.682 | 0.283 | 1.644 |
| Interaction, Factor × Sex, female |  | n.s | 1.101 | 0.935 | 1.298 |
| Estimation of present match load ^c^ | 461 | n.s | 0.877 | 0.701 | 1.098 |
| Sex, female |  | n.s | 0.815 | 0.366 | 1.818 |
| Interaction, Factor × Sex, female |  | n.s | 1.087 | 0.857 | 1.380 |
| Good balance between training/match load and recovery ^d^ | 462 | n.s | 1.094 | 0.906 | 1.320 |
| Sex, female |  | n.s | 2.093 | 0.859 | 5.096 |
| Interaction, Factor × Sex, female |  | n.s | 0.875 | 0.711 | 1.077 |
| *CI, confidence interval.*  *^*^ Based on Poisson regression on number of new injuries during the season, with the natural logarithm of total exposure hours during the season entered as an offset, and with team entered as a random factor, using variance component as covariance structure, and intervention entered as a fixed factor in addition to each potential baseline risk factor, and the 2-way interaction between each factor and sex;* ^†^ *Ankle, knee, posterior thigh, or hip/groin injury;* ^§^ *p-value of main fixed effect*  ^a^ *1 = extremely likely, 7 = extremely unlikely*; ^b^ *1 = little importance, 7 = great importance*; ^c^ *1 = extremely low, 7 = extremely high*; ^d^ *1 = strongly disagree, 7 = strongly agree* | | | | | |
